# The Use of Commas in Secondary-Education Students and Its Relationship with Reading Comprehension: The Case of Spanish

**DOI:** 10.3390/brainsci12111564

**Published:** 2022-11-17

**Authors:** Ana Marcet, Verónica Moreno, Carmen Rodríguez-Gonzalo, Manuel Perea

**Affiliations:** 1 Departamento de Didáctica de la Lengua y la Literatura, Universitat de València, 46010 Valencia, Spain; 2 Departamento de Pedagogía y Didáctica de las Ciencias Sociales, la Lengua y la Literatura, Universitat Jaume I, 12006 Castellón de la Plana, Spain; 3 Departamento de Metodología de las Ciencias del Comportamiento and ERI-Lectura, Universitat de València, 46010 Valencia, Spain; 4 Centro de Investigación Nebrija en Cognición (CINC), Universidad Nebrija, 28015 Madrid, Spain

**Keywords:** spelling, commas, secondary education, reading comprehension, didactics

## Abstract

The correct use of punctuation marks in secondary-school students is essential for the comprehension of written texts and, therefore, for the students’ academic success. However, the examination of this issue has often been overlooked in the literature. In the present study, we focused on the progression of comma usage (i.e., a punctuation mark that is often challenging to master) and examined its relation to reading comprehension. A sample of first- and fourth-year secondary-education students from Spain (N = 115) punctuated brief texts in which commas had been previously omitted. The original texts included various types of mandatory commas in Spanish. We also obtained a reading comprehension score from a standardized reading test. Results show that secondary-education students often did not correctly place the commas, with first-year secondary-education students only succeeding in the correct placement of commas 54.5% of the time in (Year 8 in the UK system). This figure increased to 80.9% in fourth-year secondary-education students (Year 11 in the UK system). As a control, this figure rose to 91.5% in first-year university students. Critically, better comprehenders were the ones with better use of punctuation marks (*r* = 0.33). These results are useful for developing teaching methods to improve writing/reading skills in secondary-school students.

## 1. Introduction

Punctuation marks are formal elements that explicitly delimit and indicate the extra- and intratextual relations in a written sentence (see [1] for a history of the evolution of punctuation marks). Punctuation marks have several functions. On the one hand, they act as intratextual markers of syntagmatic relations (e.g., compare “Peter loves cooking, his family and watching movies” vs. “Peter loves cooking his family and watching movies”). On the other hand, they delimit the scope of a paragraph or a text, indicating the textual and inferential relations that readers must make to understand the meaning of the text. Hence, punctuation marks are essential to establish the comprehension of a text by influencing the syntactic (a delimiting function), semantic (an indicating function), and pragmatic (a disambiguating function) levels [2].

Notably, the correct use of punctuation marks is relevant not only from a linguistic perspective but also for reading comprehension. Punctuation is an essential mechanism in textual macrostructure, providing the keys for correctly interpreting content and defining the discourse’s structure and meaning [3]. Furthermore, punctuation marks also indicate when to pause, how to adapt the tone of your voice and other important information. Thus, using appropriate punctuation marks is a marker of good writing and reading skills. Although some authors emphasize the role of punctuation marks as links between orality and writing (e.g., see [4]), punctuation marks are exclusive codes of the norms of the written language. Each punctuation mark contains its own meaning and is used in restricted contexts following the criteria developed in the grammar of each language.

In the present study, we focused on one punctuation mark, the comma. As Frask [5] pointed out, “this punctuation mark is frequently used and very frequently used wrongly” (p. 12). Given that we collected the empirical data in Spanish, it is convenient to indicate the definition of the comma in the agency that regulates the norms of Spanish, namely, the Royal Academy of Spanish [6]:

“*punctuation mark (,) that normally indicates the existence of a brief pause within an utterance. It is attached to the word or sign that precedes it and separated by a space from the word or sign that follows it. Its presence does not always respond to the need to pause in reading and vice versa; there are short pauses in reading that should not be marked graphically by commas. Although in some cases, the use of the comma in a certain place may depend on the writer’s intention, some commas must be present in the sentence so that it can be correctly read and interpreted*”.

Most research on the use of commas in Spanish is framed within the structuralist perspective, which focuses on the categorization of the use of each punctuation mark [7,8,9] or on the consequences of the misapplication of linguistic regulations in the written code [10,11]. In addition to assessing the proper use of commas, other proposals also include a didactic proposal that allows rethinking the teaching of the comma in the educational system. In recent years, the preeminence of the cognitive paradigm has made it possible to consider the comma as a critical element in discourse. The relevance of the role of the comma as an inferential key to interpreting the author’s discursive intention explains the interest of applied studies on the normative and differentiated uses of the comma in the educational context.

The present research had two main goals. Firstly, we examined whether Spanish secondary-education students (Year 8–11 in the UK system), who have studied the linguistic norm since elementary school, use commas appropriately according to the linguistic norm in Spanish (i.e., mandatory commas). As a control, we also tested a group of first-year university students. Secondly, under the assumption that mastering the punctuation norms involves being aware of the structure of the sentences, we tested the association between the students’ knowledge of the use of commas and their reading comprehension scores. The underlying rationale of this examination is that knowing when to use commas is key to establishing proper textual relationships, as reflected by reading comprehension. The final aim of our study was to use the answers to these two questions as a preliminary framework for a didactic approach to teaching this punctuation mark. Before introducing the details of the study, we now review the relatively scarce literature on this topic, with a particular focus on previous research in Spanish.

In Spanish, Roselló [12] compared the use of punctuation marks by a group of secondary-education students (third course of secondary education (Year 10 in the UK system) and first year of bachiller (General Certificate of Secondary Education) by analyzing their written texts at the beginning and end of the academic year. When presented with a series of sentences, the students had to indicate the correct vs. incorrect use of punctuation marks in those sentences according to the linguistic rules in Spanish. Roselló [12] found that the comma was the punctuation mark with the highest rate of errors. Among these errors, the bracketing comma was the most problematic (e.g., “Juan, nuestro profesor de lengua, fue nuestro guía en el viaje”. [John, our language teacher, was our guide on the trip.] was often written without the corresponding commas). Likewise, Julio Chitiva and Castro Robles [13] examined the use of punctuation marks in a group of 15–16-year-old students. Seventy-five percent of the students claimed to use the comma habitually in their writings but also acknowledged that around 60% of the time, they did not check afterward that their usage of commas corresponded to the linguistic norms. These issues also arise for university students. Rodríguez Muñoz and Ridao Rodrigo [14] analyzed 128 writings of first-year university students in forums for two courses. The comma presented a percentage of use of only 47%. The preeminence of comma use was also observed in a study conducted by Sánchez’s [15], who analyzed 60 essays written by first-year Colombian university students. The results showed that the comma was the most frequently used punctuation mark (62% of the total) and that the errors in its use occurred especially in the case of bracketing commas and syntagmas.

The above-cited studies focused on assessing students’ use of punctuation marks, such as the comma, through descriptive analysis of various written productions. Although this is a relevant approach, these studies do not allow us to delve into the differentiated function and implications derived from poor knowledge of these linguistic signs. Another procedure is to use texts or sentences in which the students have to place the correct commas. This was the approach followed by Popic [16] with Slovenian students enrolled in secondary education and the first year of university. Popic [16] created a survey containing 35 sentences, and the students had to place the comma correctly. The students correctly placed only 31% of the commas. This high error rate was interpreted as being due to the multiple uses of the comma and the little teaching time devoted to it in secondary education.

There have also been a few studies that have examined the role of commas (and other punctuation marks) when reading aloud in Spanish. In their standardized battery of reading for secondary-education students (PROLEC-SE-R), Cuetos et al. [17] included a subtest on punctuation marks, in which students had to read aloud a text containing various punctuation marks (e.g., commas, periods, colons, hyphens, interrogation marks, exclamation marks). While this subtest had relatively low correlations with the other subtests, it was associated, at *r* = 0.30, with academic success (measured as the student’s average grade) in the first year of secondary education (Year 8 in the UK system); this correlation decreased to 0.10 in the fourth year of secondary education. Likewise, using the PROLEC-SE-R test, Álvarez Cañizo et al. [18] found an association between reading comprehension and several measures of reading fluency when reading aloud (e.g., appropriate pauses after a comma). On a related note, other studies have also analyzed how commas affect intonation when reading aloud in Spanish. Jordan et al. [19] examined the prosodic differences in reading in children with and without language disorders in Spanish. They found that children with language disorders take unnecessary pauses in reading, not following the textual guidelines marked by punctuation marks. While these findings suggest an association between the usage of punctuation marks and reading comprehension when reading aloud, it is critical to examine whether this association also occurs in a silent reading scenario (i.e., the most common scenario when reading a text).

To our knowledge, no prior studies have examined the links between reading comprehension and comma use during silent reading in Spanish. The present study examined the progression of the usage of commas by secondary-education students in Spanish and its relationship with reading comprehension. Thus, the present study would offer useful information on whether the association between the use of commas and reading comprehension is related to syntax, and not just to prosody. The ultimate goal was to have valuable data that could offer an informed, pedagogical proposal for teaching a punctuation mark (i.e., the comma) that is often wrongly used.

The study consisted of two phases. Firstly, the students received two texts from the PROLEC-SE-R test (i.e., a standardized reading test for secondary-education students in Spanish, [17]), which were presented consecutively. One text was expository, and the other was narrative. The students had five minutes to read each text, and then they answered ten multiple-choice questions. Secondly, the students received four one-page written texts. They were told that all commas had been omitted and that their task was to punctuate the text following the punctuation norms. To create the materials, we created four texts that included sentences requiring commas according to the linguistic norms in Spanish, and their location was the only correct possible choice. We employed five types of sentences that required commas. Each text included two sentences of each type: (1) bracketing commas (“Antonio Pérez, nuestro profesor de educación física, escribe poesía en su tiempo libre”. [Antonio Pérez, our physical education teacher, writes poetry in his spare time.]); (2) connective commas (“En general, la reunión del sábado fue un éxito”. [All in all, Saturday’s meeting was a success.]); (3) listing (numbering) commas (“Mis frutas favoritas son las manzanas, las naranjas y las peras” [My favorite fruits are apples, oranges and pears.]); (4) adversative commas (“Pedro escribe bastante bien, aunque puede redactar mucho mejor”. [Peter writes quite well, although he can write much better.]); and (5) concessive commas (“Por mucho que insistas, ella no te dará las notas hasta mañana”. [No matter how much you insist, she will not tell you the grades until tomorrow.]). The key difference between adversative and concessive sentences is that adversative sentences establish an opposition between propositions. In contrast, concessive sentences express a relation of logical dependence of the subordinate concerning the main clause. Each text also included several sentences that did not need a comma. After reading each of the four texts, the students had to answer two comprehension questions to ensure that they had read and understood each text adequately. We also conducted exploratory analyses to test: (1) the progression of the use of each type of comma, and (2) whether reading comprehension was particularly related to one of these types of commas; note that it may be more difficult to master the use of bracketing than listing commas (see [12,15]). For comparison purposes, we recruited a sample of first-year university students. The rationale was that this group would reflect the potential progress after secondary education; note that in the Spanish system, there are two academic years (Years 12–13 in the UK system) between secondary education and university.

## 2. Method

### 2.1. Participants

The participants were recruited from a public secondary school in a middle-class area of Valencia (Spain). Sixty-two students were in their first year of secondary school (Year 8 in the British system; range: 12–13 years), and sixty-four were in their fourth year (Year 11 in the British system; range: 15–16 years). All tutors/participants provided informed consent before starting the study, and the Research Ethics Committee of the Universitat de València approved this study. All participants were native speakers of Spanish with no history of reading or learning difficulties. The proposed final sample size was 50 in each group in the registration of this research proposal. Still, we assumed that a small percentage of participants might not finish the task, so the initial sample was slightly greater than 50. In addition, we recruited 65 first-year university students from the School of Education at the Universitat de València.

### 2.2. Materials

We created four brief texts comprising approximately half a page, requiring ten commas each. In each text, there were two instances of each comma type, including sentences with bracketing commas (e.g., “Su madre, nutricionista de profesión, trabaja desde casa”. [His mother, a nutritionist by profession, works from home.]), listing commas (“Cada año tiene que comprarse zapatos, pantalones y camisas”. [Every year he has to buy shoes, pants and shirts.]), connective commas (e.g., “Asimismo, los animales tienen una gran importancia en sus cuadros”. [Likewise, animals are of great importance in his paintings.]), concessive commas (e.g., “Se autoabastecen de carne y huevos, aunque su mayor orgullo es el huerto”. [They are self-sufficient in meat and eggs, although their greatest pride is their vegetable garden.]), and adversative commas (e.g., “A pesar de ser una vivienda pequeña, el espacio está bien organizado”. [Despite being a small house, the space is well-organized.]). We also made two open comprehension questions for each text. The four texts are available in the OSF link indicated in the Data Availability Statement subsection. In addition, we employed two texts from the PROLEC-SE-R battery [17] to obtain the participants’ reading comprehension scores: “The platypus” (expository) and “The betrayal” (narrative); two texts were used to obtain more stable comprehension scores.

### 2.3. Procedure

The study took place in the classroom. The students were told that the study consisted of two phases. In the first phase (reading comprehension subset of the PROLEC-SE-R battery), they had to read two brief texts for comprehension for 5 min, one at a time, and answer ten four-choice questions relative to the text (first with an expository text and then with a narrative text). In the second phase, the students were presented with four brief texts, one at a time, in which the commas had been omitted; note that other punctuation marks were present in the texts (e.g., periods, semicolons). The participant’s task was to place the commas according to the Spanish norms where necessary. They were asked two comprehension questions immediately after each text, on the same page. The entire session took approximately 35 min to complete.

## 3. Results

A small subset of the participants had incomplete data (i.e., did not complete the four texts with commas or the two comprehension tests). As a result, the final sample size of secondary-education students was 115 (56 in the first year and 59 in the fourth year), and the number of first-year university students was 61. In a small percentage of cases (24 occurrences overall), the participants placed a comma where there should not be one; including these trials did not essentially affect the results.

The first goal of the present study was to examine the progression of the use of various types of commas in first-year and fourth-year secondary-education students presented with a series of texts in which the commas had been omitted. Overall, results show a relatively low usage of commas in first-year secondary-education students; commas were placed only 54.5% of the time. This percentage increased to 80.4% in fourth-year secondary-education students. As a control, the parallel number for first-year university students was 91.5%.

**Figure 1:**
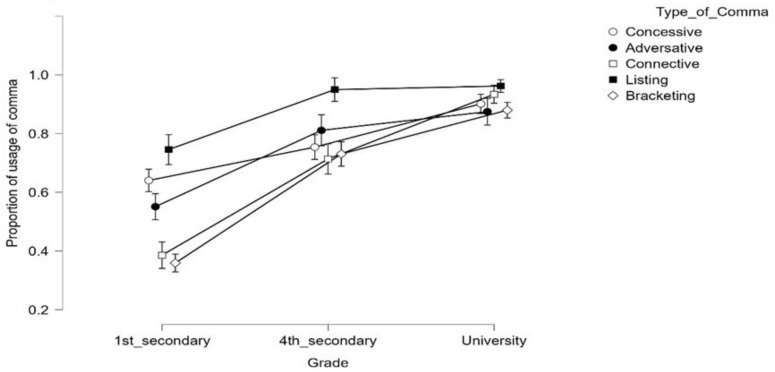
The proportion of use of the various types of commas in first-year students of secondary education, fourth-year students of secondary education, and first-year university students. For each condition, the bars represent the 95% confidence intervals.

To further examine this pattern, we tested whether the type of comma modulated the use of mandatory commas across the different grades. Although this analysis is certainly exploratory (e.g., the sentences in the texts may differ in several potentially relevant factors, so some caution is necessary), observing the progression of the use of the different types of commas in the three groups of participants may provide relevant information (see Figure 1). An analysis of variance on the proportion of comma usage with grade as a between-subjects factor and type of comma as a repeated-measure factor showed not only the main effects of grade, *F*(2, 174) = 77.664, *p* < 0.001, and type of comma, *F*(4, 692) = 69.850, *p* < 0.001, but also a sizeable interaction between the two factors, *F*(8, 692) = 16.234, *p* < 0.001. The full ANOVA is presented in a link provided in the Data Availability subsection. This interaction revealed that the use of listing commas was already relatively high (74.6%) in first-year secondary-school students. Its use increased close to the ceiling (95.0%) in their fourth year of secondary school; it was 96.2% for first-year university students. Instead, the use of bracketing and connective commas was challenging for first-year secondary-education students (35.9% and 38.6%, respectively). Notably, their use went up to 73.1% and 71.4%, respectively, for fourth-year students; these numbers increased to 88.0% and 93.4%, respectively, for university students. Somewhat in the middle, adversative and concessive commas were indicated 55.1% and 64.1% of the time, respectively, in first-year students of secondary education; this increased to 81.1% and 75.4% in the fourth year. For university students, these numbers were 87.5% and 90.2%, respectively.

**Figure 2:**
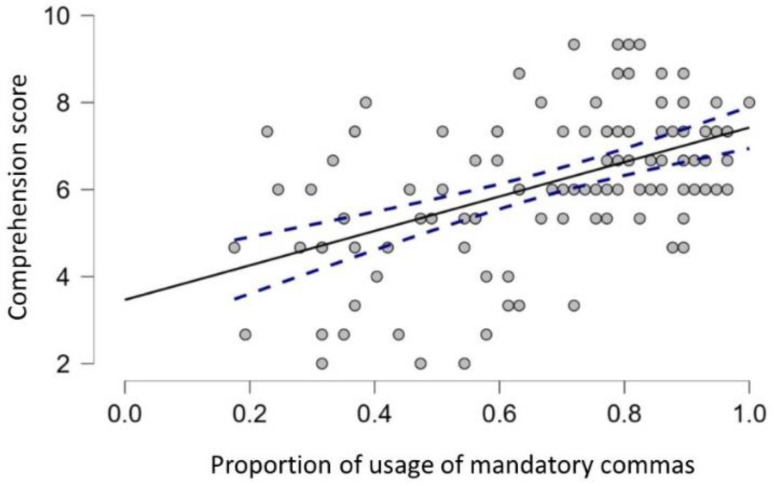
Scatter plot showing the regression line (and its 95% confidence interval) between the secondary-education students’ use of commas (proportion of correct use in the sentences) and their reading comprehension scores.

The second question of the present study was whether the use of commas in secondary-school students is associated with reading comprehension (see Figure 2 for a scatter plot with the association). (Note that the PROLEC-SE-R test was designed for secondary-school students only, not for university students.) Results show a moderate association between the use of commas and reading comprehension in secondary-education students, *r* = 0.332, *p* < 0.001, after controlling for grade (first year vs. fourth year of secondary education). The correlation index without controlling for grade was *r* = 0.586. This correlation was higher for first-year than for fourth-year secondary-education students (*r* = 0.394 vs. *r* = 0.198, respectively); note, however, that the scores of fourth-year secondary-education students were less heterogeneous (and with overall better performance), and this could have decreased the value of the Pearson correlation index. As an aside, we conducted exploratory analyses that tested the association of each type of comma with reading comprehension scores. The association was numerically similar for all types of commas (all *r*s > 0.265, all *p*s < 0.004) except for the concessive commas (*r* = 0.131, *p* = 0.163). This association was numerically greatest for the bracketing commas, *r* = 0.323; the corresponding *r* values for the listing, connective, and adversative commas were 0.309, 0.266, and 0.266, respectively. While suggestive, these small differences should be considered with caution.

## 4. Discussion

The correct use of punctuation marks by secondary-school students is essential for the comprehension of written texts and, therefore, for the academic success of students. Indeed, the incorrect use of punctuation marks may indicate poor writing and reading abilities. However, this issue has often been overlooked in the literature on reading. In the present study, we focused on the use of a frequent—and often challenging to master—punctuation mark: the comma. We examined this issue by recruiting a sample of secondary-education students in Spain. The students had to place the appropriate commas in four texts in which the commas had been omitted. Results show that first-year secondary-school students (Year 8 in the UK system) only included the mandatory commas 54.5% of the time (Year 8 in the UK system). This was especially the case for bracketing and connective commas. This figure increased to 80.9% for fourth-year secondary-education students (Year 11 in the UK system). Overall, the best performance was seen for the listing comma; its use was near the ceiling for fourth-year secondary-education students (Year 11 in the UK system).

Importantly, reading comprehension as assessed with a standardized test was associated with the use of commas (overall, the Pearson correlation coefficient was 0.332). This finding reveals that the correct use of punctuation marks is related to reading comprehension (see [20] for evidence when teaching Spanish as a foreign language). The essential role of the comma in correctly interpreting an utterance is evident in studies on natural language processing. The logic is that the versatility of use and implications of this punctuation mark make direct coding difficult (see [21]). We conducted exploratory analyses to test whether some types of commas were more associated with reading comprehension. We found that this association was (numerically) highest in the case of the bracketing comma; note that, as shown in Figure 2, these commas produced a high error rate for first-year secondary-school students. One explanation is that bracketing commas are challenging because they require placing two commas (i.e., opening and closing commas). This difficulty relies on converting the language’s absolute complexity into relative complexity, which can be explained by the Optimality Theory [22]. In this framework, the user would simplify the linguistic complexity (the opening comma) by understanding that the inferential relationship between the terms is explicit but would not omit the closing comma because, if they did so, the prosodic patterns of the utterance would be altered. As Llisterri et al. [23] suggested, prosodic boundaries cannot be inferred directly in these cases; it would be necessary for readers to perform a syntactic analysis to disambiguate the paralinguistic pattern. Of note, bracketing commas are not challenging only because two commas must be inserted. First-year secondary-education students also performed poorly on connective commas, which require only one placement.

While beyond the scope of the present study, future research should examine if readers produce different prosodic patterns depending on the commas present in the sentences or if, for example, in the case of omission, they maintain the prosodic structure of the subordinate clause. The necessary disambiguating action implies that the reader must perform a conscious linguistic examination of the utterance. This is consistent with the view that the incorrect use of commas is related to difficulties in establishing the syntactic connections of the text [24], so the reader cannot identify the antecedents of relations correctly. We acknowledge that additional research is necessary to provide a more fine-grained delimitation of the role of commas in sentence reading. One such option is to register the participants’ eye movements when reading sentences with a particular focus on the regions around the omitted commas (see [25] for a similar approach when examining the role of accent marks during reading). A complementary option is to explore the electrophysiological signature of the presence vs. absence of the mandatory commas (e.g., see [26] for early evidence of the role of commas in English with event-related potentials during reading), which could be modulated by reading ability.

We now briefly examine how the present findings could serve as a preliminary framework for future pedagogical approaches in secondary school. We have shown that: (1) secondary-education students often struggle with the placement of commas in sentences, and (2) the correct use of commas is associated with reading comprehension in a silent reading scenario. At this educational level, most research has focused on testing whether students have learned the grammatical rules of comma usage (e.g., [13,14]). We believe that teaching the use of commas should not be limited to learning grammatical rules but should also be related to their function. The aim would be to better understand the textual relationships established by commas. Teaching approaches should be based on an inferential approach that allows students to grasp the intratextual relationships indicated by commas. This idea is based on the co-constructing knowledge framework, where learning cannot be separated from language [27]. The link between reading comprehension and the correct interpretation of commas is related to understanding the text’s syntactic structure. This methodology requires a specific emphasis on metalinguistic reflection (focus on form in Anglophone approaches). In other words, to understand a text, it is not enough to merely understand the general meaning or semantic clarifications linked to the signifier–meaning relationship. When trying to comprehend a text, students must decode the position of the comma and infer its implication in relation to the syntax, thus learning to interpret the meaning that the author provided to the writing [28]. In this way, students can internalize and understand the comma’s function in ambiguous contexts, such as in explanatory sentences. Future studies with pedagogical applications should corroborate whether learning the function of the comma, focused on reading comprehension, improves the levels of knowledge and use of the comma. Another avenue of research relates to the potential links between the use of punctuation in L1 and L2; note that some of the rules are different across languages (e.g., the serial (Oxford) comma).

## Data Availability

The four texts, together with the data, scripts and results, are available at the following link: https://osf.io/6cmhk/?view_only=28e74d6c22814c26bcaf6bd3232c3e00 (accessed on 20 September 2022).
